# Management of Pulmonary Hemorrhage Complicating Pulmonary Thromboendarterectomy

**DOI:** 10.3389/fmed.2018.00326

**Published:** 2018-11-21

**Authors:** Adam A. Dalia, Scott Streckenbach, Mike Andrawes, Richard Channick, Cameron Wright, Michael Fitzsimons

**Affiliations:** Department of Anesthesiology, Massachusetts General Hospital, Harvard Medical School, Boston, MA, United States

**Keywords:** pulmonary hemorrhage, chronic thromboembolic pulmonary hypertension, airway management, thromboendarterectomy, CTEPH, cardiac anesthesia, double lumen tube

## Abstract

Airway management during pulmonary thromboendarterectomy (PTE) can prove challenging, especially in the face of unexpected intraoperative pulmonary hemorrhage. Utilization of proper airway equipment on induction is crucial for the successful management of intraoperative pulmonary hemorrhage. Our case series describes the preoperative risk factors that can lead to intraoperative pulmonary hemorrhage, the preinduction airway equipment considerations for PTE, and the intraoperative management of pulmonary hemorrhage. We summarize the lessons learned at our institution from four cases of post perfusion pulmonary hemorrhage.

## Introduction

Pulmonary thromboendarterectomy (PTE) for chronic thromboembolic pulmonary hypertension (CTEPH) is an increasingly common surgical procedure (1). Pulmonary hemorrhage associated with PTE during the perioperative period is a rare, but life threatening complication (2–9). The management of such a complication has been discussed, but very little has been published on the appropriate techniques for airway management in the setting of hemorrhage associated with PTE (5–7).

## Case series

Four cases of pulmonary hemorrhage have occurred among 140 patients undergoing PTE (2.9%) at our institution. We describe a case series of intraoperative pulmonary hemorrhage and the challenges of airway management in this setting. We review the current literature and make several recommendations from our experience.

## Case 1

A 71-year-old man with a history of chronic lower extremity deep venous thrombosis (DVT), pulmonary emboli, and pulmonary hypertension on home oxygen and Ventavis® (iloprost) presented for PTE. During the rewarming period after PTE, bright red blood was noted in the endotracheal tube. A bronchoscope was used to suction 750 ml of blood from the airway. After an unsuccessful attempt to place a left sided Arndt endobronchial blocker (Cook Medical, Bloomington, IN,USA) with a standard adult bronchoscope the decision was made to attempt to exchange the single lumen 8 mm endotracheal tube (ETT) to a 41F left sided double lumen tube (DLT). The initial DLT exchange over a tube exchanger was unsuccessful. The patient was extubated and a DLT 41F left sided tube was placed under direct visual laryngoscopy and position confirmed with a 5.2 mm distal tip diameter bronchoscope. Approximately 1L of blood was suctioned from the left endobronchial lumen. Single right lung ventilation was initiated and separation from cardiopulmonary bypass (CPB) was successful. Prior to transfer to the cardiac surgical intensive care unit (CSICU), the left lung bleeding slowed and two-lung ventilation was initiated. Respiratory failure in the post-operative period resulted in a need for veno-venous extracorporeal membrane oxygenation (VV ECMO). The bleeding stopped overnight and the DLT was exchanged for a standard ETT. The patient developed acute respiratory distress syndrome (ARDS) and multi-organ failure. Comfort measures only were initiated, and the patient expired on postoperative day (POD) 9.

## Case 2

A 71-year-old man with a history of CTEPH presented for PTE. During the rewarming process following PTE, bright red blood was noted in the airway. Bronchoscopy revealed bleeding from the right lung. Placement of a right sided Arndt endobronchial blocker (Cook medical, Bloomington, IN, USA) was attempted but was unsuccessful due to copious bleeding. The 8.0 mm single lumen ETT was exchanged for a size 9 mm single lumen ETT. Then a right sided 9Fr Arndt endobronchial blocker was placed to contain the bleeding with the aid of a 6.0 mm outer diameter therapeutic bronchoscope. The weaning from CPB continued successfully, and the patient was transferred to the ICU. The patient's postoperative course was complicated by renal failure, reintubation for hemoptysis, and poor neurologic recovery. Due to the multiple complications, comfort only measures were initiated and the patient expired on POD 20.

## Case 3

A 54-year-old woman with past medical history of COPD and CTEPH presented for PTE. During the CPB weaning process high airway pressures were noted along with bright red blood in the airway. Bronchoscopy noted significant blood coming from the right bronchus. Initial placement of an Arndt endobronchial blocker was attempted but unsuccessful. The 7.5 mm ETT was then exchanged for a size 8.5 mm ETT with the use of a Glidescope® video laryngoscopy (Verathon, Seattle, Washington, USA). After placement of the 8.5 mm ETT, a right sided Arndt endobronchial blocker was effectively positioned with a 6.0 mm outer diameter therapeutic bronchoscope. Significant pulmonary hypertension and hypoxemia remained and the decision was made to place her on venoarterial (VA) ECMO. The patient had a protracted complicated postoperative course. She was eventually weaned from ECMO but developed renal failure and ARDS. In conjunction with the patient's family wishes, comfort only measures were initiated and the patient expired on POD 12.

## Case 4

A 65-year-old woman with history of obesity, OSA, and CTEPH presented for PTE. During rewarming, bright red blood was noted in the ETT. Bronchoscopy demonstrated bleeding from the left lung. Initial placement of an Arndt endobronchial blocker and ETT exchange over a stylet were unsuccessful. The patient was extubated and direct laryngoscopy was utilized to place an 8.5 mm ETT. A 9Fr Arndt endobronchial blocker was then placed in the distal left main bronchus with the aid of a 6.0 mm outer diameter therapeutic bronchoscope. After removal of blood from the airway with bronchoscopy, two lung ventilation with low tidal volumes and PEEP was instituted. CPB could not be weaned due to poor oxygenation and hemodynamic instability so VA ECMO was instituted. Her postoperative course in the ICU was complicated by ongoing renal, hepatic, and right ventricular failure as well as ARDS. She expressed a desire for comfort measures only and expired on POD 25.

**Figure 1:**
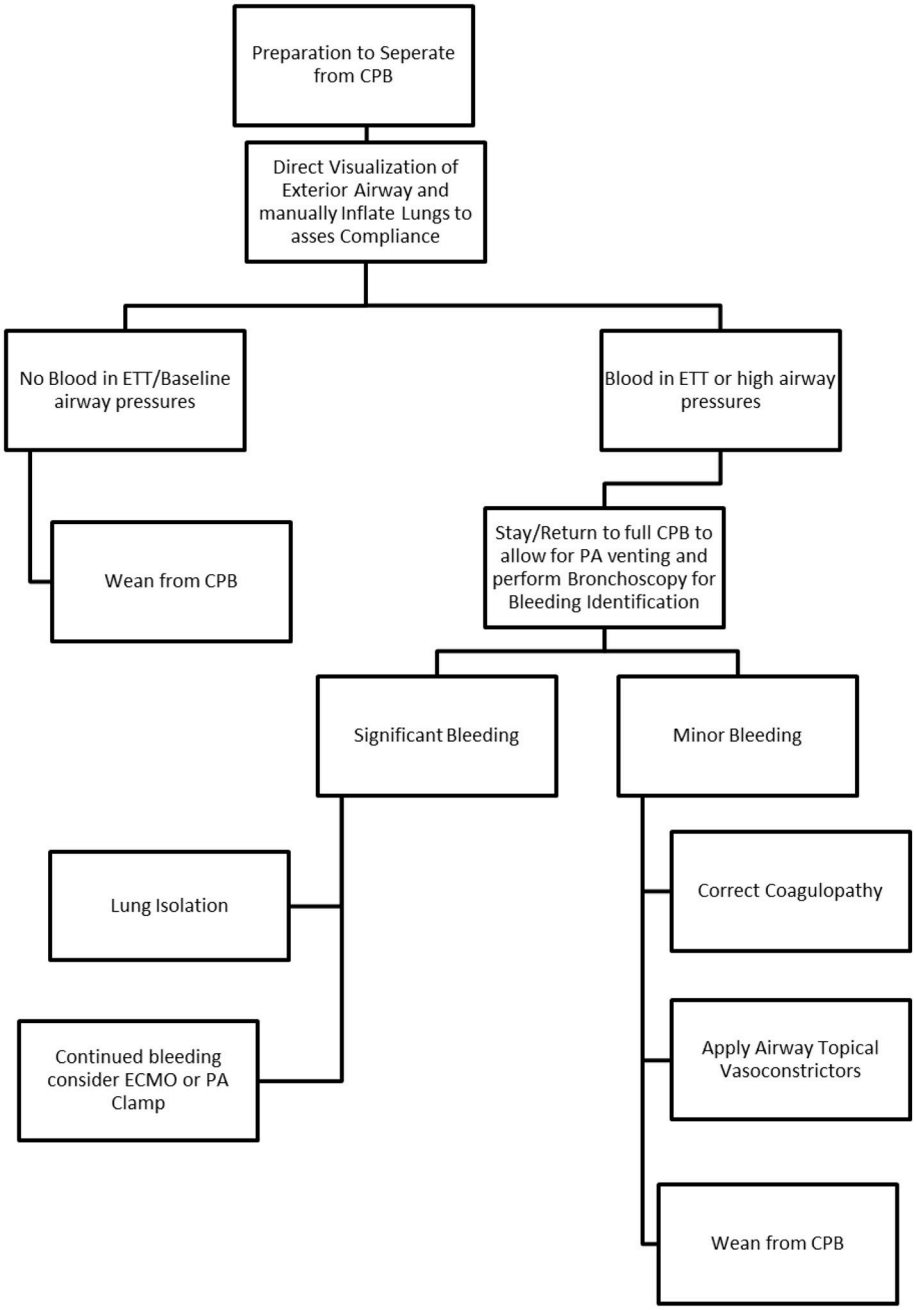
Algorithm for CTEPH Post Cardiopulmonary Bypass Management.

A synopsis of clinical factors is seen in Table 1 and relevant clinical algorithm in Figure 1.

**Table 1:** Pre-operative Status.

**Case**	**Jamieson type**	**Hemodynamics**	**Echocardiography**	**Airway on induction**	**Airway after bleeding**
1	Type 3	RA 19 mmHg PA 76/32 (mean 51) mmHg CO 5.5 L/min	Dilated RV Hypokinetic RV Severe TR EF 55%	8 mm SLT	41Fr Left DLT
2	Type 3	RVSP 77 mmHg	Severely dilated RV Severe TR EF 55%	8mm SLT	9mm SLT and 9Fr BB
3	Type 2–3	RA 10 mmHg PA 61/28 (mean 40) mmHg	Mildly decreased RV function Normal EF Moderate TR	7.5 mm SLT	8.5 mm SLT and 9Fr BB
4	Type 3–4	PA 90/49 (mean 62) mmHg PCWP 10 mmHg CO 3.9 L/min	Severe RV hypokinesis Mild TR EF 60–65%	7.5 mm SLT	8.5 mm SLT and 9Fr BB

*RA, Right atrium; PA, Pulmonary artery; CO, Cardiac output; RV, Right ventricle; EF, Ejection fraction; TR, Tricuspid regurgitation; SLT, Single Lumen Tube; DLT, Double Lumen Tube; BB, Bronchial Blocker*.

## Discussion

As of 2016 mortality associated with surgical correction of CTEPH has been described by Madini et al. to be 4.4 and 2.2% depending on the time frame sampled and without specific mention of pulmonary bleeding (10). Significant pulmonary hemorrhage is reported to occur in approximately 1% of PTE cases and is associated with a mortality of nearly 70% (11). The most common source of bleeding is a tear or disruption in the intima of the pulmonary artery generally in more distal segmental arteries where surgical dissection is more complicated (6, 12). The challenge of airway management in the setting of pulmonary hemorrhage is complicated. In addition to the challenges of oxygenation and ventilation, complications such as air embolization have been reported (13).

Risk factors for hemorrhage associated with PTE have not been well studied; Manecke et al reported three cases out of 600 procedures (0.5%) and suggested that more friable tissue, older age, residual pulmonary hypertension, and Jamieson classification appeared to be associated factors (4, 12). The Jamieson classification describes the pathologic location and significance of the pulmonary vascular disease (Table 2) (12). Disease that is distal in the pulmonary vessels (Jamieson 3–4) is more difficult if not impossible to remove, and the progressive fragility of the tissue predisposes to injury (11, 12). Patients that have higher classification disease have more residual pulmonary hypertension and a one-month mortality of 14.3% (12). Additionally, PA wall calcification from long-standing occlusion may be an additional risk factor for hemorrhage, as it may commit the surgeon to a deeper dissection plane.

**Table 2:** Jamieson Classification (11, 14, 15).

**Type**	**Description**	**Prevalence (%)**	**1-Month Postop Mortality (%)**
1	Organized or fresh clot in the major-vessel lobar or main pulmonary arteries visible upon opening the pulmonary artery	37–38	1.3
2	No major-vessel clot visible, but intimal thickening and fibrosis present proximal to segmental arteries	40–49	2.5
3	Very distal fibrosis, webbing, and intimal thickening within distal segmental arteries without visible thrombus	12–18	13.2
4	Microscopic distal vessel disease with no evidence of thromboembolic material	1.6–3	14.3

Management of the airway presented different challenges in each of the 4 cases. Three of the cases involved replacing the single lumen ETT with a larger size to facilitate use of an endobronchial blocker, while the first case involved placement of a DLT. Continued bleeding and the diffuse coating of the airway in blood obscured the identification of the site of bleeding which impeded placement of the endobronchial blocker. We learned that use of a standard adult bronchoscope does not provide enough suctioning ability to clear large amounts of blood and that the therapeutic bronchoscope is much more efficient, but requires a large ETT.

Several modes of airway management have been reported in the setting of pulmonary hemorrhage during PTE including the Univent™ (Teleflex, Morrisville, NC, USA), Arndt Endobronchial blocker (Cook medical, Bloomington, IN, USA), Rusch® EZ Blocker™ (Teleflex, Morrisville, NC, USA), and double lumen endotracheal tube (Medtronic, Minneapolis, MN, USA) (2–4, 7). Cronin et al reported the use of a Univent ETT along with a bicaval dual lumen VV ECMO and suggested a treatment algorithm (2).

Anesthesiologists facing this challenge may reflexively select a DLT for emergent management. The DLT may be placed initially without the use of a fiberoptic bronchoscopy and allows visualization via bronchoscopy afterwards (16). The DLT is more secure in the bronchus and permits a degree of suctioning to remove blood and clot from the airway but does not allow ready suctioning with an adult bronchoscope (5, 16). The larger size of the DLT may predispose to vocal cord injury and is usually replaced by a single lumen ETT later in recovery (17).

An endobronchial blocker has the advantage of avoiding the need to change the ETT. The endobronchial blocker may also be positioned in the specific lower lobe rather than blocking an entire main bronchus. The disadvantages of an endobronchial blocker include less reliable stability within the lobe making it easier to dislodge, and provides less effective isolation (18). The endobronchial blocker must be placed through a large ETT (19).

An intermediate third option is a Rusch® EZ Blocker™ (Teleflex, Morrisville, NC, USA). This is a 7Fr endobronchial blocker with a single length catheter that has two distal ends with inflatable cuffs that is placed through a single lumen ETT and sits at the main carina. With two distal ends each seated in a different main bronchus, either cuff may be inflated depending on which lobe needs isolation. The advantages of this type of blocker are quick engagement, good seating in the bronchus (20), less sore throat than a DLT (21), and easy placement through a preexisting airway (18). The disadvantages include inability for segmental lobe isolation, which may make ventilation and oxygenation more difficult.

In our institution, the available fiber optic intubation scope (STORZ, Germany, Tuttlingen) has a 5.2 mm distal tip outer diameter while the therapeutic bronchoscope (Olympus, Tokyo, Japan) has a 6.0 mm distal tip outer diameter. When pulmonary hemorrhage occurs it is ideal to be able to diagnose the location of the bleeding, and as such the capability to appropriately suction the airway requires a larger sized bronchoscope with a large suction channel. Taking into consideration that a bronchoscope must be used in conjunction with placing some sort of endobronchial blocker the internal diameter of the endotracheal tube becomes crucial. Available adult endobronchial blockers range from 2.33 to 3 mm in outer diameter and adult bronchoscope outer diameters range from 5.2 to 6 mm; it becomes clear that no <8.5 mm single lumen ETT should be placed for PTE's. This 8.5 mm internal diameter single lumen endotracheal tube can accommodate the manipulation of a bronchial blocker, bronchoscope, all the while continuing to oxygenate, and ventilate the patient.

Our cases share the common factors that initiation of ventilation during rewarming following DHCA while still on CPB yielded high airway pressure and bleeding. Bronchoscopy was able help rule out other causes of high airway pressures including non-cardiogenic pulmonary edema, reperfusion edema, and transfusion related acute lung injury (TRALI) (22). Fiberoptic bronchoscopy was also utilized to localize bleeding, the source being the left lung in 2 cases and the right lung in the remaining 2 cases.

In each of the 4 cases, airway manipulation occurred during cardiopulmonary bypass (CPB). Bypass does provide certain advantages during complicated airway manipulation including the ability to divert blood away from the lungs while supporting all oxygenation and ventilation. However, uncontrolled bleeding into the lung parenchyma may become so significant that sequestration leads to circuit fluid depletion. CPB may also allow intermittent flow to the lungs to facilitate accurate localization of bleeding and thus appropriate use of either a targeted bronchial blocker or DLT prior to separation from bypass. This preemptive response to diffuse pulmonary bleeding while on CPB can reduce the amount of blood in the airway and the extent of airway destruction once attempting to separate from bypass.

Additional techniques to help control pulmonary bleeding after endarterectomy include correction of coagulopathy, topical vasoconstrictors, and specific ventilator management, some suggesting the initial use of positive end expiratory pressure (PEEP) to help oxygenate and localize the source of bleeding (5, 8). With our patients, various ventilation strategies were applied ranging from pressure control to ventilation control to a pressure limiting volume guaranteed mode with varying rates of success during the brisk hemorrhaging, as such ventilation likely plays a minor role in the immediate management of pulmonary hemorrhage. Topical vasoconstrictors can play a role in mitigating pulmonary bleeding with phenylephrine and vasopressin saline lavage administered via the ETT if readily available (4). Reddy et al reported the successful use of overnight pulmonary artery clamping to stop bleeding (14). Given the right circumstances and operating room capability the possibility of immediate pulmonary angiography and coil embolism could help mitigate further pulmonary bleeding, although both technically challenging and resource demanding this option is not practical in our current healthcare landscape. With refractory bleeding, not amenable to treatments described above the use of both VV and VA ECMO have been reported extensively (15, 23). Utilizing ECMO in this scenario allows for proper oxygenation as well as time for pulmonary intimal damage to repair (2). As most of these interventions don't necessarily correct the damaged intima or vessel but rather rely on a temporizing measure to allow for self-healing, ECMO is a viable option to help improve oxygenation while allowing the damaged pulmonary vasculature and intima time to heal.

## Conclusion

Pulmonary hemorrhage associated with PTE is uncommon but carries a high mortality rate (15). Once such hemorrhage is identified, airway management during a time of active bleeding can be difficult if not impossible. The key issues to management are maintenance of oxygenation and ventilation, identification of site of hemorrhage, isolation of the segment, and appropriate post-operative care. A key teaching point from our CTEPH cases is that the airway device placed on induction should be viewed as a possible conduit for a bronchial blocker and/or therapeutic bronchoscope in the event of pulmonary hemorrhage. We recommend a size 8.5 mm internal diameter or larger single lumen endotracheal tube to be placed on induction, and a bronchial blocker and therapeutic bronchoscope should be readily available during the rewarming process after endarterectomy. Our institutional recommendations are summarized in the included algorithm (Figure 1). Controlling and containing the hemorrhage as soon as possible preserves the remaining lung and may allow successful ventilator weaning and salvage of the patient.

## Author contributions

AD, SS, MA, RC, CW, and MF helped in caring for the patients in this report as well as collecting and analyzing patient factors, drafting, and editing this manuscript. MF was the senior author with final editorial standards.

### Conflict of interest statement

The authors declare that the research was conducted in the absence of any commercial or financial relationships that could be construed as a potential conflict of interest.
